# Quantification of cerebral perfusion and cerebrovascular reserve using Turbo‐QUASAR arterial spin labeling MRI†

**DOI:** 10.1002/mrm.27956

**Published:** 2019-09-12

**Authors:** Moss Y. Zhao, Lena Václavů, Esben T. Petersen, Bart J. Biemond, Magdalena J. Sokolska, Yuriko Suzuki, David L. Thomas, Aart J. Nederveen, Michael A. Chappell

**Affiliations:** ^1^ Institute of Biomedical Engineering University of Oxford Oxford United Kingdom; ^2^ Wellcome Centre for Integrative Neuroimaging FMRIB Centre Nuffield Department of Clinical Neurosciences University of Oxford Oxford United Kingdom; ^3^ Amsterdam UMC University of Amsterdam, Radiology and Nuclear Medicine Amsterdam Netherlands; ^4^ Danish Research Centre for Magnetic Resonance Centre for Functional and Diagnostic Imaging and Research Copenhagen University Hospital Hvidovre Hvidovre Denmark; ^5^ Centre for Magnetic Resonance, DTU Elektro Technical University of Denmark Kgs Lyngby Denmark; ^6^ Amsterdam UMC University of Amsterdam, Haematology, Internal Medicine Amsterdam Netherlands; ^7^ Medical Physics and Biomedical Engineering University College London Hospitals London United Kingdom; ^8^ Neuroradiological Academic Unit Department of Brain Repair and Rehabilitation UCL Queen Square Institute of Neurology University College London London United Kingdom; ^9^ Leonard Wolfson Experimental Neurology Centre UCL Queen Square Institute of Neurology University College London London United Kingdom

**Keywords:** arterial spin labeling, cerebral blood flow, cerebrovascular reserve, pseudo‐continuous arterial spin labeling, pulsed arterial spin labeling, Turbo‐QUASAR

## Abstract

**Purpose:**

To compare cerebral blood flow (CBF) and cerebrovascular reserve (CVR) quantification from Turbo‐QUASAR (quantitative signal targeting with alternating radiofrequency labeling of arterial regions) arterial spin labeling (ASL) and single post‐labeling delay pseudo‐continuous ASL (PCASL).

**Methods:**

A model‐based method was developed to quantify CBF and arterial transit time (ATT) from Turbo‐QUASAR, including a correction for magnetization transfer effects caused by the repeated labeling pulses. Simulations were performed to assess the accuracy of the model‐based method. Data from an in vivo experiment conducted on a healthy cohort were retrospectively analyzed to compare the CBF and CVR (induced by acetazolamide) measurement from Turbo‐QUASAR and PCASL on the basis of global and regional differences. The quality of the two ASL data sets was examined using the coefficient of variation (CoV).

**Results:**

The model‐based method for Turbo‐QUASAR was accurate for CBF estimation (relative error was 8% for signal‐to‐noise ratio = 5) in simulations if the bolus duration was known. In the in vivo experiment, the mean global CVR estimated by Turbo‐QUASAR and PCASL was between 63% and 64% and not significantly different. Although global CBF values of the two ASL techniques were not significantly different, regional CBF differences were found in deep gray matter in both pre‐ and postacetazolamide conditions. The CoV of Turbo‐QUASAR data was significantly higher than PCASL.

**Conclusion:**

Both ASL techniques were effective for quantifying CBF and CVR, despite the regional differences observed. Although CBF estimated from Turbo‐QUASAR demonstrated a higher variability than PCASL, Turbo‐QUASAR offers the advantage of being able to measure and control for variation in ATT.

## 1 INTRODUCTION

Arterial spin labeling (ASL) is an MRI technique that allows the quantification of cerebral blood flow (CBF).^1^ Under certain acquisition conditions, it is also possible to quantify other parameters, such as arterial transit time (ATT) and arterial blood volume.^2^ Although pseudo‐continuous ASL (PCASL) with a single post‐labeling delay (PLD) has been recommended as the standard implementation,^3^ it can be sensitive to changes in ATT, which varies in different regions of the brain, potentially affecting the precise quantification of CBF.^4^ Given that ASL does not expose the subject to ionizing radiation and is readily repeatable and noninvasive, it can also be used to measure cerebrovascular reserve (CVR), an important biomarker reflecting the maximal change in CBF that can be achieved relative to the baseline CBF in response to a vasoactive stimulus.^5^ However, if the recommended ASL implementation is utilized in CVR experiments, in which data need to be collected under different induced blood flow conditions, CBF and CVR might be wrongly estimated because of the change in ATT caused by the varying arterial flow velocity.^6^ For example, in a cerebrovascular reactivity study using multi‐PLD PCASL, the researchers demonstrated a significant increase in CBF between 3.5% and 27.8% and a significant decrease in ATT between 3.3% and 7.7% during hypercarbia, suggesting that variations in ATT should be controlled for the accurate quantification of CBF in CVR studies.^7^ Additionally, the inversion efficiency of PCASL is sensitive to changes in velocity of the inflowing blood in the labeling plane,^8^ potentially leading to further inaccuracy in the quantified CBF and CVR. Thus, both ATT and inversion efficiency might be confounds to CVR quantification,^9^ making single‐PLD PCASL a potentially less desirable tool. In contrast to PCASL, the inversion efficiency of pulsed ASL (PASL) is less sensitive to changes in blood flow velocity,^10^ and when a multi‐PLD or multi‐TI (inversion time) imaging technique is utilized to simultaneously estimate ATT, its variation can be corrected for.^11^ However, typical PASL labeling gives rise to far lower signal‐to‐noise ratio (SNR) than PCASL attributable to the shorter bolus duration as well as the influence of T_1_ decay on the label.^12^

Recently, a novel PASL technique, dubbed Turbo‐QUASAR (quantitative signal targeting with alternating radiofrequency labeling of arterial regions), has been developed with the aim of improving the SNR of conventional PASL techniques, by creating a series of boluses of labeled blood‐water in a single ASL experiment, while retaining the ability to estimate ATT by utilizing a multi‐TI Look‐Locker readout.^13^ Although preliminary work on Turbo‐QUASAR showed comparable SNR with PCASL and full brain coverage,^13^ it remains unknown whether Turbo‐QUASAR provides effective and accurate CBF measurement. In particular, because of the repeated radiofrequency (RF) pulses in the label and control experiments of Turbo‐QUASAR, magnetization transfer (MT) effects could potentially affect the estimation accuracy of CBF.^14^, ^15^ Given that Turbo‐QUASAR combines the features of PASL and multi‐TI acquisition, it may potentially be a suitable ASL‐based technique for CVR quantification where substantial variation in flow velocity and ATT might be confounding because of the administration of a vasoactive stimulus.

The aim of this work was to (1) develop a model‐based method for CBF and ATT quantification for Turbo‐QUASAR and (2) investigate the application of Turbo‐QUASAR for CVR measurement. Simulations were performed to evaluate the estimation accuracy and sensitivity of parameter estimation to variations in ATT and bolus duration, as might be expected within a study population and with variation in blood flow velocity. Data from an in vivo experiment collected on a healthy cohort were retrospectively analyzed to compare the CBF and CVR (induced by acetazolamide administration) measurement between Turbo‐QUASAR and single‐PLD PCASL on a full brain and regional basis. The quality of the PCASL and Turbo‐QUASAR data was assessed by the coefficient of variation (CoV) of the estimated CBF values.

## 2 THEORY

### 2.1 Turbo‐QUASAR sequence

**Figure 1:**
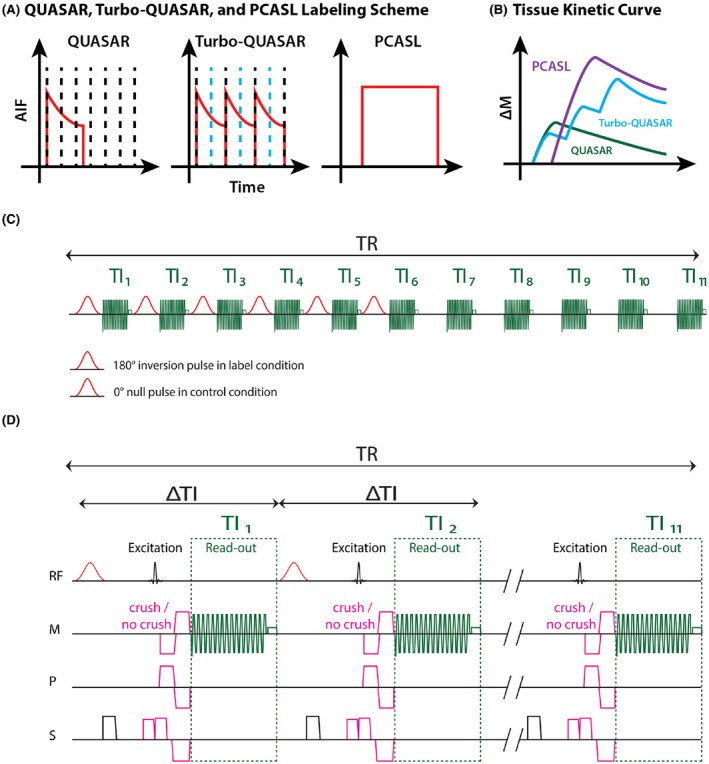
Turbo‐QUASAR techniques and pulse sequence diagrams. (A) In QUASAR, only one short‐duration bolus is created (shown by the typical PASL AIF curve in red) and a Look‐Locker readout is performed to acquire multi‐PLD (black dash lines) ASL data. In Turbo‐QUASAR, labeling (blue dash line) and acquisition (black dash line) are interleaved and multiple boluses are created. Hence, a summation of multiple boluses creates a higher SNR. In PCASL, the inflowing spins are labeled continuously, creating a longer duration bolus with the typical “rectangular” PCASL AIF. (B) Tissue kinetic curve for QUASAR ASL (green), Turbo‐QUASAR (blue), and PCASL (purple). (C) In Turbo‐QUASAR, each TR consists of six label/control pulses and 11 TIs. Null pulses were used in the control condition to minimize the impact of MT effects. (D) Presaturation, label/control pulses, crushing gradients in three directions, and Look‐Locker readout

As an improvement of the QUASAR method,^2^, ^16^ Turbo‐QUASAR was designed to overcome the low SNR of PASL when compared to PCASL by applying a series of labeling pulses to create a longer effective bolus duration.^13^ These labeling pulses were inserted between readout (also replacing the QUIPPS II pulse used in QUASAR) to maintain the magnetization level of the tracer for a longer period than conventional PASL techniques, as shown in Figure 1, thus increasing the overall SNR of the resulting ASL data. Figure 1A shows the different labeling techniques and the associated arterial input function for QUASAR, Turbo‐QUASAR, and PCASL. In essence, the total bolus duration of each Turbo‐QUASAR experiment is the summation of the duration of each sub‐bolus created by each RF pulse. Because of the multiple labeling pulses and the interleaving of labeling and imaging, the QUIPPS II style bolus saturation pulse used in QUASAR cannot be applied in Turbo‐QUASAR, which might lead to an uncertain duration for each of the individual boluses. As in QUASAR, the readout scheme of Turbo‐QUASAR is Look‐Locker, but the time between each successive readout (ΔTI) was chosen to be longer than QUASAR in order to allow enough time for the inflowing blood‐water inverted by one labeling pulse to leave the labeling region before the subsequent labeling pulse. While maintaining the same time for acquiring each slice, this not only allows more slices to be acquired to reach a nearly full brain coverage, but also reduces rapid signal decay from the repeated Look‐Locker excitations. Supporting Information Figure S1B shows the kinetic curve of the signal in the tissue from QUASAR, Turbo‐QUASAR, and PCASL. Given that Turbo‐QUASAR includes multiple labeling pulses, the total effective bolus duration (and SNR) is much larger than QUASAR. Consistent with the original Turbo‐QUASAR work,^13^ we refer to the bolus duration as the total effective duration of all labeling pulses in a Turbo‐QUASAR experiment. We define sub‐bolus duration (SBD) as the duration of the tracer created by a single labeling pulse. Given that each labeling pulse is applied exactly between each successive Look‐Locker acquisition, the SBD should be identical to the time between each readout (ΔTI of 600 ms in this study). This can be achieved by optimizing the width of the labeling slab and considering the flow velocity such that all labeled spins would flow out of the labeling region before the next labeling pulse is applied. Under such conditions, the bolus duration of Turbo‐QUASAR can be determined by summing up the duration of all the sub‐boluses. Under nonideal conditions (e.g., suboptimal choice of labeling thickness for a given arterial blood velocity), the SBD might be shorter than expected. Another distinctive feature of Turbo‐QUASAR is that slices are acquired from different locations in each repeat to achieve full‐brain coverage while keeping the same effective temporal resolution with QUASAR for the accurate estimation of ATT. The detailed description of this strategy is explained in Supporting Information Figure S1.

### 2.2 Calibration

The absolute quantification of CBF requires the measurement of equilibrium magnetization of the arterial blood (M_0a_), which can be computed from the equilibrium magnetization of the tissue (M_0t_). Similar to QUASAR, M_0t_ can be estimated by fitting the control images to a saturation recovery model.^16^ In Turbo‐QUASAR, given that the labeling pulses are applied repeatedly between the Look‐Locker readout, the recovery of the static tissue signal is influenced by two effects as shown in Supporting Information Figure S2: (1) affected by both Look‐Locker readout and cumulative MT effects attributed to the repeatedly labeling pulse; (2) affected by Look‐Locker readout alone. Although the correction for the Look‐Locker effect follows the same principle in QUASAR,^16^ a correction for the MT effects is needed for estimating M_0t_ in Turbo‐QUASAR. Specifically, the magnetization of the spins in the static tissue during the readout can be modeled by a modified saturation recovery model that separates signals affected by both MT effects and Look‐Locker readout during the period where labeling pulses are being applied ($t\leq t_{I}$), and by Look‐Locker readout alone ($t>t_{I}$) (Equation 1):

(1) $$M\left(t\right)=\left\{\begin{array}{c} M_{0t\_MT}^{\prime }\;\cdot \;A\;\cdot \;\left(1-e^{-\frac{t}{T_{1t\_MT}^{\prime }}}\right),\;0<t\leq t_{I}\\ M_{0t}^{\prime }-\left(M_{0t}^{\prime }-M_{I}\right)\;\cdot \;A\;\cdot \;e^{-\frac{t-t_{I}}{T_{1t}^{\prime }}},\;t>t_{I} \end{array}\right.$$

The definitions of the terms used here can be found in Table 1. While it is expected that there would be voxel‐wise variation in T_1_ and therefore $T_{1t}^{\prime }$ in the brain, $T_{1t\_MT}^{\prime }$ would also be expected to vary with proximity to the labeling region, as shown in Supporting Information Figure S2. Subsequently, M_0t_ and T_1t_ (without the influence of MT and Look‐Locker effects) can be estimated from $M_{0t}^{\prime }$ and $T_{1t}^{\prime }$ by applying the correction method on Look‐Locker effects used in QUASAR.^16^

**Table 1:** Parameter definitions

Term	Definition
*A*	Saturation efficiency
α	Inversion efficiency
$c_{i}\left(t\right)$	AIF of the *ith* sub‐bolus
$\varepsilon \left(v\right)$	T_1_ corrected efficiency for a spin with velocity v
*f*	CBF
*L*	Width of the labeling slab in Turbo‐QUASAR
*m*_i_(*t*)	Magnetization decay function of the *ith* sub‐bolus
*M*_0_ *_a_*	Equilibrium magnetization of the arterial blood
*M*_0_ *_t_*	Equilibrium magnetization of the tissue
$M_{0t}^{\prime }$	Equilibrium magnetization of the tissue affected by Look‐Locker effects
$M_{0t\_\mathrm{MT}}^{\prime }$	Equilibrium magnetization of the tissue affected by MT and Look‐Locker effects
$\Delta M\left(t\right)$	Tissue signal of the ASL data
$\Delta M_{i}\left(t\right)$	Tissue signal of the *ith* sub‐bolus
*M_I_*	Signal at $t_{I}$
ATT	Arterial transit time
ΔTI	Time between each readout
τ	Sub‐bolus duration (SBD)
λ	Blood‐water partition coefficient
*T*_1_ *_a_*	T_1_ relaxation time of the arterial blood
$T_{1t}^{\prime }$	T_1_ relaxation time of the tissue affected by Look‐Locker effects
$T_{1t\_\mathrm{MT}}^{\prime }$	T_1_ relaxation time of the tissue affected by MT and Look‐Locker effects
$t_{I}$	Time at which MT effects terminate
*v*	Flow velocity
*v_max_*	Maximum velocity in the center of the laminar flow profile

### 2.3 Tissue kinetic model

The ASL difference signal of Turbo‐QUASAR can be considered as the summation of the signal from each sub‐bolus (as illustrated in Figure 1) that can be described using the general kinetic model.^17^ The definitions of the terms used can be found in Table 1. The arterial input function (AIF; $c_{i}\left(t\right)$) of the *ith* sub‐bolus is given by Equation 2:

(2) $$c_{i}\left(t\right)=\left\{\begin{array}{c} 0,\;0<t<i\cdot \Delta TI+ATT\\ e^{-\frac{t-i\cdot \Delta TI}{T_{1a}}},\;i\cdot \Delta TI+ATT\leq t<i\cdot \Delta TI+ATT+\tau \\ 0,\;i\cdot \Delta TI+ATT+\tau \leq t \end{array}\right.$$

The residue function remains the same as in QUASAR.^16^ For the magnetization decay function $m_{i}\left(t\right)$, both the Look‐Locker readout and MT effects affect the decay of the label in tissue. For the *ith* sub‐bolus that has arrived at the tissue region, the decay function can be approximated by Equation 3:

(3) $$m_{i}\left(t\right)=\left\{\begin{array}{c} e^{-\frac{t}{T_{1t\_MT}^{\prime }}},\;0<t<\text{max}\left(0,t_{I}-i\cdot \Delta TI-ATT\right)\\ e^{-\frac{t}{T_{1t}^{\prime }}},\;t_{I}\leq t \end{array}\right.$$

that is, labeled blood‐water present in the tissue while labeling is continuing subject to MT effects and thus experiences an accelerated relaxation modeled by the $T_{1t\_MT}^{\prime }$ time constant that can be estimated voxelwise from the control images. Incorporating the analytical solution of the tissue kinetic model for PASL,^18^ the tissue signal of each sub‐bolus can be expressed by Equation 4:

(4) $$\Delta M_{i}\left(t\right)=\left\{\begin{array}{c} 0,\;0\;<t\;<i\cdot \Delta TI+ATT\\ \frac{F\cdot e^{\frac{-(t-i\cdot \Delta TI)}{T_{1t\_MT}^{\prime }}}}{R_{\mathrm{MT}}^{\prime }}\cdot \left(e^{R_{\mathrm{MT}}^{\prime }\cdot \left(t-i\cdot \Delta TI\right)}-e^{R^{\prime }\cdot ATT}\right),\;i\cdot \Delta TI+\Delta t\leq t\;<i\cdot \Delta TI+ATT+\tau \\ \frac{F\cdot e^{\frac{-(t-i\cdot \Delta TI)}{T_{1t\_MT}^{\prime }}}}{R_{\mathrm{MT}}^{\prime }}\cdot \left(e^{R_{\mathrm{MT}}^{\prime }\cdot \left(\Delta t+\tau \right)}-e^{R^{\prime }\cdot ATT}\right),\;i\cdot \Delta TI+\Delta t+\tau \leq t\;<t_{I}-i\cdot \Delta TI-ATT-\tau \\ \frac{F{\cdot e}^{\frac{-(t-i\cdot \Delta TI)}{T_{1t}^{\prime }}}}{R^{\prime }}\cdot \left(e^{R^{\prime }\cdot \left(\Delta t+\tau \right)}-e^{R^{\prime }\cdot ATT}\right),\;t_{I}-i\cdot \Delta TI-ATT-\tau \leq t \end{array}\right.$$

where $R_{\mathrm{MT}}^{\prime }=1/T_{1t\_MT}^{\prime }-1/T_{1\alpha },\;R^{\prime }=1/T_{1t}^{\prime }-1/T_{1\alpha },\;F=2\cdot \alpha \cdot M_{0,\alpha }\cdot f/\lambda$. The four components of $\Delta M_{i}\left(t\right)$ represent: (1) before the arrival of the the *ith* sub‐bolus (2) from the arrival of the the *ith* sub‐bolus to the end of the the *ith* sub‐bolus, in which the signal is influenced by MT and Look‐Locker effects (3) from the end of the *ith* sub‐bolus to MT effects terminate (the final sub‐bolus does not have this part) (4) after the MT effects terminate, in which the signal is only affected by Look‐Locker readout pulses. Finally, the acquired signal $\Delta M\left(t\right)$ of Turbo‐QUASAR is the summation of the signal of each sub‐bolus $\Delta M\left(t\right)=\sum \Delta M_{i}\left(t\right)$.

## 3 METHODS

### 3.1 Simulation experiments

Three simulation experiments were conducted to investigate the accuracy of the model‐based method for Turbo‐QUASAR under various flow conditions. Simulated Turbo‐QUASAR difference data were created using the parameters listed in Table 2. A reference signal value was assumed to be the highest signal intensity of the kinetic curve generated by these parameters with one sub‐bolus. Noise was added to the simulated data assuming white noise according to $N\left(0,\;SD\right)$ where SD=Referencesignal/SNR with 1000 realizations.

**Table 2:** Parameter values used in simulations and in vivo experiments

Parameter	Value
All simulation experiments	
CBF (mL/100 g/min)	60
ATT (ms)	700
TI (ms)	40, 640, …, 6040
Tissue T_1_ (ms)	1300
Arterial blood T_1_ (ms)	1650
Simulation experiment 1	
No. of sub‐boluses	1, 2, …, 7
SBD (τ) (ms)	600
SNR	10, INF
Simulation experiment 2	
No. of sub‐boluses	7
SBD (τ) (ms)	300, 400, 500, 600
Variations added to SBD (ms)	N (0, 10)
SNR	5, 10, 50, 100
Simulation experiment 3	
No. of boluses	7
SBD (τ) (ms)	600
SNR	5, 10, 50, 100
ATT (ms)	800, 1000, 1200
In vivo experiment using Turbo‐QUASAR	
CBF (mL/100 g/min)	(0, 10^12^)^a^
ATT (ms)	(700, 10^–1^)^a^
Tissue T_1_ (ms)	Estimated from the control data
Arterial blood T_1_ (ms)	1650
Total number of sub‐boluses	6
SBD (τ) (ms)	Estimated from PC‐MRI data
Blood‐water partition coefficient	0.98
Inversion efficiency	0.91
In vivo experiment using PCASL	
CBF (mL/100 g/min)	(0, 10^12^)^a^
ATT (ms)	1300
PLD (ms)	1800
Bolus duration (ms)	1800
Tissue T_1_ (ms)	1300
Arterial blood T_1_ (ms)	1650
Blood‐water partition coefficient	0.98
Inversion efficiency	estimated from PC‐MRI data

a Values indicate the mean and standard deviation of the Gaussian prior used in the model fitting.

Abbreviation: INF, infinity.

Experiment 1 examined the accuracy of CBF estimation using model‐based analysis of Turbo‐QUASAR under different SNRs. The level of SNR was manipulated by (1) varying the number of sub‐boluses from 1 to 7 in order to vary bolus duration and (2) varying the amount of noise by changing the SD. For analysis, the SBD (τ) was set to be 600 ms (same as in the in vivo experiment) and not estimated by the model‐based analysis. The mean and the standard deviation of the estimated CBF were computed.

Experiment 2 investigated the accuracy of the model‐based method under various flow conditions. As discussed, the SBD in Turbo‐QUASAR is designed to be equal to the time period between each successive RF pulse under normal flow velocity and laminar flow. In conditions where the flow becomes more rapid, however, the effective SBD would decrease. To model the rapid flow, the simulated SBD (τ) was set to 300, 400, 500, and 600 ms plus a random variation drawn from a normal distribution, N(0, 10 ms). Given that the time between each readout (∆TI) used in the in vivo data was 600 ms, it was not possible for SBD to exceed 600 ms, thus longer SBDs were not considered in the simulation experiments. Data were generated over a range of SNRs: 5, 10, 50, and 100. CBF, ATT, and SBD were estimated using the model‐based technique in four ways:

1. *Default SBD Step 1:* CBF and ATT were estimated assuming the default value of SBD (τ=600ms, without the added random variation).
2. *Default SBD Step 2:* CBF, ATT, and SBD were all estimated using the results from step 1 as the priors for the model‐fitting process and assuming a constant value of SBD (τ=600ms, without the added random variation).
3. *Corrected SBD Step 1:* CBF and ATT were estimated using the simulated SBD values (τ=300,400,500,or600ms without the added random variation).
4. *Corrected SBD Step 2:* CBF, ATT, and SBD were all estimated using the results from step 3 as the priors for the model‐fitting process and using the simulated SBD values (τ=300,400,500,or600ms without the added random variation).

Techniques 1 and 2 aimed to investigate the case where SBD was assumed to be the default (theoretical) value. Techniques 3 and 4 aimed to investigate the case where the measured flow velocity from the phase contrast MRI (PC‐MRI) data provided an independent estimation of the SBD that is close (but not identical) to the simulated SBD value. For all experiments, the relative error was computed by dividing the absolute value of the difference between the estimated value and the simulated value by the simulated value using the following equation (Equation 5):

(5) $$Error_{\mathrm{relative}}=\frac{\left|X_{\mathrm{estimated}}-X_{\mathrm{simulated}}\right|}{X_{\mathrm{simulated}}}\times 100\%$$

The mean and the standard deviation of the relative errors were reported.

Experiment 3 investigated the accuracy of ATT estimation using Turbo‐QUASAR when a prolonged ATT was expected. In this case, the simulated ATT values were set to be 0.8, 1.0, and 1.2 seconds and CBF and SBD were 60 mL/100 g/min and 0.6 seconds, respectively. Noise was added using the SNR values of 5, 10, 50, and 100. The mean and standard deviation of the estimated ATT were computed.

### 3.2 Healthy cohort data

All procedures were approved by the local institutional review board of the Amsterdam University Medical Centers, Location Academic Medical Center, The Netherlands. The procedures were carried out according to the Declaration of Helsinki. Data were analyzed from a study in which 11 healthy volunteers (mean age 39 ± 17 years, 5 female) were included after they had given written informed consent.^19^ Exclusion criteria were contraindications to MRI and acetazolamide, history of stroke, brain injury, neurological disease, and injury that might affect cerebral autoregulation. Each subject underwent antecubital placement of a venous cannula and MRI with acetazolamide administration. Acetazolamide was administered at a dose of 16 mg/kg body weight with a maximum of 1400 mg. All images were acquired on a clinical 3.0T MR system (Ingenia; Philips Healthcare, Best, The Netherlands) with a 32‐channel receive head‐coil and body‐coil transmission.

The following MR image series were collected from each subject: (1) baseline—3D T_2_‐FLAIR (fluid‐attenuated inversion recovery), 2D PC‐MRI, Turbo‐QUASAR; (2) PCASL, acquired immediately before, during, and after acetazolamide administration; and (3) postacetazolamide administration: PCASL calibration M_0_, Turbo‐QUASAR, and 2D PC‐MRI. Table 3 lists the parameter values of the MRI data. PCASL and Turbo‐QUASAR imaging volumes were planned identically such that the middle slice of each volume corresponded to the same slice in both scans. Supporting Information Figure S1C,D shows the pulse sequence diagram of Turbo‐QUASAR. Similar to QUASAR,^2^ crushing gradients with three different directions were applied in Turbo‐QUASAR before the Look‐Locker acquisition. Data collected with the crushing gradients were considered to represent signal from the brain tissue, and data from without the crushing gradients were signals from both the tissue and the arterial blood (macrovasculature). Each repeat of Turbo‐QUASAR consisted of seven dynamics with six of them acquired using the normal flip angle (35°) and one using the low flip angle (11.7°) as described in Chappell et al.^16^ Among the six dynamics of normal flip angle, four of them were acquired with the crushing gradients and two without the crushing gradients. In the dynamics of low flip angle, crushing gradients were not used. In the in vivo Turbo‐QUASAR data of this study, two repeats were acquired, making the full data include eight dynamics with the crushing gradients and six dynamics without the crushing gradients. The order of the slices acquired in these two repeats was different, as explained in Supporting Information Figure S1. The first 35 repeats (dynamics) of PCASL were used as baseline scans. At repeat 35, acetazolamide was administered. The last 35 repeats were used as postacetazolamide scans, assuming the effect of acetazolamide reached a plateau 10 to 15 minutes after injection.^20^ The imaging slice of the PC‐MRI and the labeling plane of PCASL were planned at the same location (90 mm below the center of the PCASL imaging volumes), and it was placed perpendicular to the vessels in the neck at cervical level C1.

**Table 3:** Imaging parameters of the MRI data

Parameter	Value
3D T_2_‐FLAIR	
TE/TR (ms)	356/4800
T_2_ prep TE (ms)	125
Matrix/FOV	256 × 256 × 321/250 × 250 × 180
Voxel size (mm)	0.98 × 0.98 × 1.12
IR delay (ms)	1650
Scan duration (min)	5:07
2D PC‐MRI	
TE/TR (ms)	5.5/15
Flip angle (degrees)	15
Matrix/FOV	512 × 512/230 × 230
Voxel size (mm)	0.45 × 0.45 × 4
Scan duration (min)	1:02
Turbo‐QUASAR	
TE/TR (ms)	16/6000
TI (ms)	40, 640, …, 6340
ΔTI (ms)	600
Flip angle (degrees)	35, 11.7
Matrix/FOV	64 × 64/240 × 240
Voxel size (mm)	3.75 × 3.75 × 7
Width of labeling slab (*L*) (cm)	15
No. of sub‐boluses	6
No. of slices	15
Slice acquisition time (ms)	36
Repeats	2
Vascular crushing VENC (cm/s)	4
Scan duration (min)	3:24
PCASL	
TE/TR (ms)	14/4400
PLD (ms)	1800
Bolus duration (ms)	1800
Matrix/FOV	80 × 80/240 × 240
No. of slices	19
Slice acquisition time (ms)	42.1
Voxel size (mm)	3 × 3 × 7
Repeats	140
Scan duration (min)	20:39
PCASL M_0_	
TE/TR (ms)	14/4400
Matrix/FOV	80 × 80/240 × 240
Voxel size (mm)	3 × 3 × 7
No. of slices	19
Repeats	6
Scan duration (min)	0:26

FOV = field of view; IR = inversion recovery; VENC = velocity encoding.

### 3.3 Blood flow velocity quantification

PC‐MRI images were segmented manually to measure the average velocity of the arterial blood in each vessel in the labeling region. This included the left and right internal carotid arteries and vertebral arteries. Measuring velocity in these arteries served two purposes: (1) to estimate the bolus duration of Turbo‐QUASAR and (2) to estimate the inversion efficiency of PCASL.

### 3.4 Absolute CBF quantification of Turbo‐QUASAR

For each subject, estimation of the effective SBD of both pre‐ and postacetazolamide conditions was performed using the mean flow velocity of the arterial blood in both internal carotid arteries from the PC‐MRI data. The estimated SBD value (τ) was either (1) assumed to be equal to the time between each acquisition (ΔTI) if the estimated flow velocity was equal to or less than the velocity threshold ($v_{0}=25\;cm/s$) set for the width of the labeling slab^13^ or (2) computed using the width of the labeling slab and the mean measured flow velocity, using the following formulation (Equation 6):

(6) $$\tau =\left\{\begin{array}{c} \Delta TI,\;v\leq v_{0}\\ \frac{L}{v},\;v>v_{0} \end{array}\right.$$

Subsequently, the bolus duration of Turbo‐QUASAR of both pre‐ and postacetazolamide was computed by summing the duration of all sub‐boluses.

For each subject, CBF and ATT of Turbo‐QUASAR in both pre‐ and postacetazolamide conditions were estimated by fitting the tissue kinetic model (Equation 4) to the data of the tissue component using the spatial Variational Bayesian inference technique^21^, ^22^ and the parameter values in Table 2. All Turbo‐QUASAR control data were used in estimating the calibration M_0_ data using the technique described in the *Theory* section. The inversion efficiency and blood‐water partition coefficient were assumed to be identical to the values used in the QUASAR study (0.91 and 0.98, respectively).^23^

### 3.5 Absolute CBF quantification of PCASL

The efficiency of flow driven inversion in PCASL was simulated in Matlab (2016; The Mathworks, Inc., Natick, MA) by solving the Bloch equations using matrix representation.^24^ “Balanced” PCASL settings were: gradient (Gmax = 5.0 mT/m, Gave = 0.36 mT/m) and RF pulse train (Hanning shaped RF pulses of 0.48‐ms duration and 1.21‐ms spacing, flip angle 27.81°), consistent with the labeling train parameters implemented in the scanner. To reflect a realistic distribution of flow velocities, a laminar flow profile was assumed and appropriate velocity weighting performed as proposed by Maccotta et al^24^ (Equation 7):

(7) $$\alpha =\frac{1}{2v_{\max}^{2}}\int_{0}^{v_{\max}}v\;\cdot \;\varepsilon \left(v\right)dv$$

Simulations were performed for $v_{\max}$ values ranging from 1 to 100 cm/s with 1‐cm/s step size. Efficiency was measured at the spin location 3 cm away from the labeling plane and the time it took to cover that distance used for T_1_ relaxation correction. For each subject, the mean velocity in each vessel was computed from the acquired PC‐MRI data. The effective inversion efficiency of each subject was computed by summing the inversion efficiency of each vessel and weighted by the fraction of blood volume contributed by each vessel out of the total volume.^19^

CBF was estimated using the spatially regularized Variational Bayesian inference technique in the FSL tool BASIL^21^, ^22^ assuming a fixed ATT of 1300 ms and the estimated inversion efficiency for each subject before and after the administration of acetazolamide. The parameter values used in model fitting are shown in Table 2. For calibration, given that the TR of the M_0_ image of PCASL was shorter than 5s, the signal intensity was corrected by multiplying by a factor of $1/(1-e^{-TR/T_{1,Tissue}})$, where the $T_{1,Tissue}$ value was chosen to be 1300 ms (same with the value used in model fitting) for the whole brain.^3^ The partial volume effects on the edge of the calibration image were corrected using the erosion and extrapolation technique.^25^ Finally, the estimated CBF images were calibrated voxelwise by the corrected $M_{0a}$.

For both Turbo‐QUASAR and PCASL, CVR in each voxel was computed using the absolute CBF values of baseline and acetazolamide conditions by the following equation (Equation 8):

(8) $$CVR=\frac{CBF_{\mathrm{Acetazolamide}}-CBF_{\mathrm{Baseline}}}{CBF_{\mathrm{Baseline}}}\times 100\%$$

### 3.6 Turbo‐QUASAR and PCASL data quality assessment

The quality of the Turbo‐QUASAR and PCASL data was assessed at the baseline condition using the CoV of the estimated CBF maps. For each voxel, the Variational Bayesian inference technique models the unknown CBF as a distribution and provides an estimation of the mean and standard deviation of this distribution.^21^ The CoV was defined as the ratio between the standard deviation and the mean of the estimated CBF. The mean CoV of all the voxels was computed for the Turbo‐QUASAR and PCASL data of each subject. Only the first 25 repeats of the PCASL baseline data were used to compute the CoV in order to match the acquisition time of Turbo‐QUASAR (3.5 minutes). A low CoV value indicates that the estimated CBF has low variability and high confidence.

The SNRs of Turbo‐QUASAR and PCASL data were compared at baseline condition. For Turbo‐QUASAR, noise was estimated by computing the standard deviation of the residuals of the model‐fit across all the TIs of the in vivo data. The signal was defined as the ASL difference signal expected at TI = 3.63 seconds (maximum signal) when CBF = 60 mL/100 g/min and ATT = 0.7 seconds and using the estimated M_0_ of each subject. For PCASL, noise was estimated by computing the standard deviation of the residuals of the model fit of the first 25 repeats (matching the same acquisition time with Turbo‐QUASAR) of the in vivo data. The signal was defined as the expected ASL difference signal at PLD = 1.8 seconds by assuming CBF = 60 mL/100 g/min and ATT = 1.3 seconds and using the M_0_ value of each subject.

### 3.7 Statistical analysis

Two‐tailed paired *t* tests were conducted to compare the global and regional (voxel‐wise) CBF and CVR between the various quantification techniques. All CBF and CVR maps were transformed to the standard Montreal Neurological Institute (MNI)152 space by combining the rigid‐body transformation matrix of the ASL difference data to the T_2_‐FLAIR structural image of each subject and a second transformation matrix obtained by registering the T_2_‐FLAIR image to the MNI152 2‐mm standard brain using the FSL tool FLIRT.^26^ Given that the coverage of Turbo‐QUASAR was slightly smaller than PCASL, a mask for group analysis was created using the coverage of Turbo‐QUASAR by taking space covered by all subjects. Before each *t* test, all images were smoothed using a Gaussian spatial filter of 5‐mm full width at half maximum. Tests were conducted using the FSL tool RANDOMISE with 5000 permutations^27^, ^28^ under the null hypothesis that the CBF or CVR values were the same. Family‐wise error rate was corrected using the method developed by Holmes et al,^29^ and the corrected *P* value was recorded and thresholded at 0.05 for significance. Voxel‐wise CBF and CVR differences in standard space were computed for each test.

## 4 RESULTS

### 4.1 Simulated experiments

**Figure 2:**
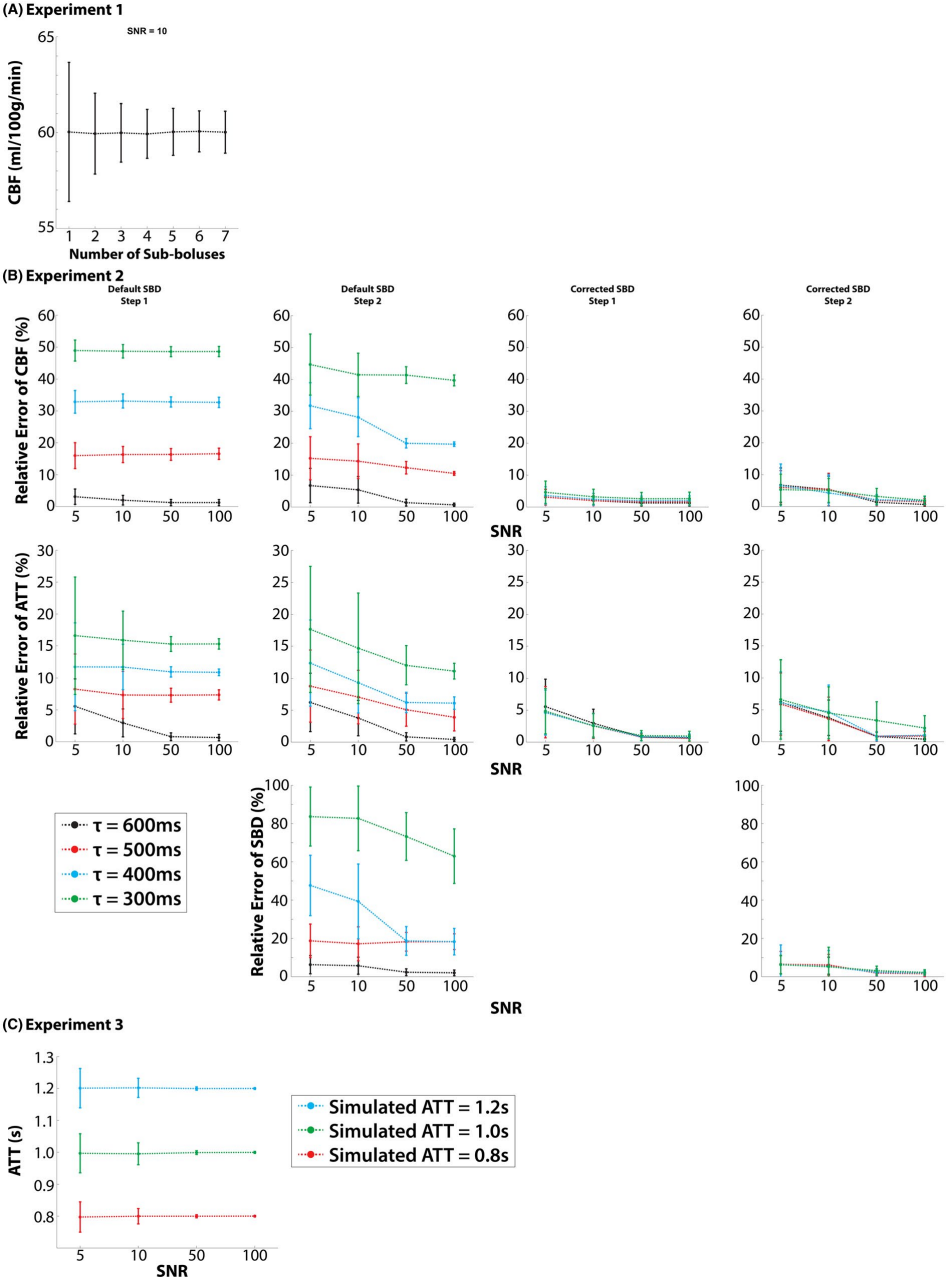
Results of simulated experiments. (A) Experiment 1: estimated CBF values for the different number of sub‐boluses (simulated CBF = 60 mL/100 g/min). (B) Experiment 2: relative error between estimated and simulated values when the simulated SBD was varied. In default SBD, the errors were low only when the SBD was equal to the global value. In corrected SBD, the accuracy of the estimation was predominantly affected by the SNR. (C) The estimation of ATT was only affected by SNR

Figure 2A shows the estimated CBF using the model‐based method from the simulated Turbo‐QUASAR data with a different number of sub‐boluses. The estimated mean CBF was consistently accurate at 60 mL/100 g/min for SNR = 10. With more sub‐boluses created, which increased the signal, the standard deviation of the estimated CBF reduced, but it plateaued after four sub‐boluses. Figure 2B shows the relative error between the estimated parameters and the simulated values when the simulated SBD was varied. Overall, the model‐based technique was accurate in estimating CBF and ATT if the correct SBD was supplied to the model‐fitting algorithm. In default SBD, where we attempted to estimate the effective SBD directly from the data in model fitting assuming a default τ value of 600 ms as the prior, the results were only accurate if the prior matched the simulated value (black line). Model fitting alone was not able to correct fully for variation in SBD, although it offered some improvement at higher SNR than simply taking the default value alone (i.e., improvement from step 1 to step 2). In corrected SBD, where the prior of the SBD was a closer match to the simulated SBD, greater accuracy could be achieved. Figure 2C shows the estimated ATT values at different SNRs. The accuracy of ATT estimation was only slightly affected by SNR.

### 4.2 CBF and CVR quantification of healthy cohort data

**Figure 3:**
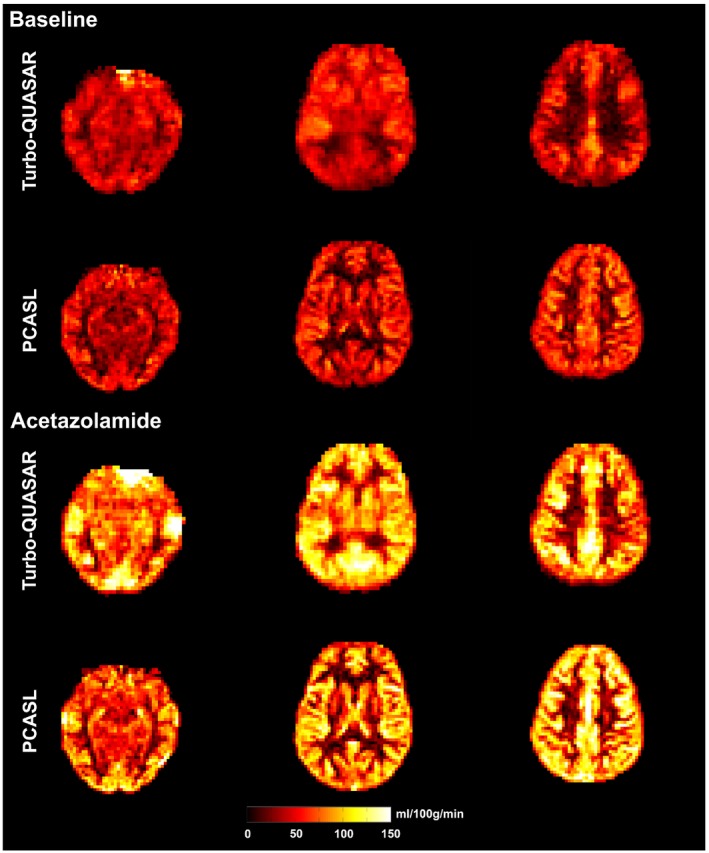
CBF maps of an example subject before and after the administration of acetazolamide. Overall, CBF increased after the administration of acetazolamide

**Figure 4:**
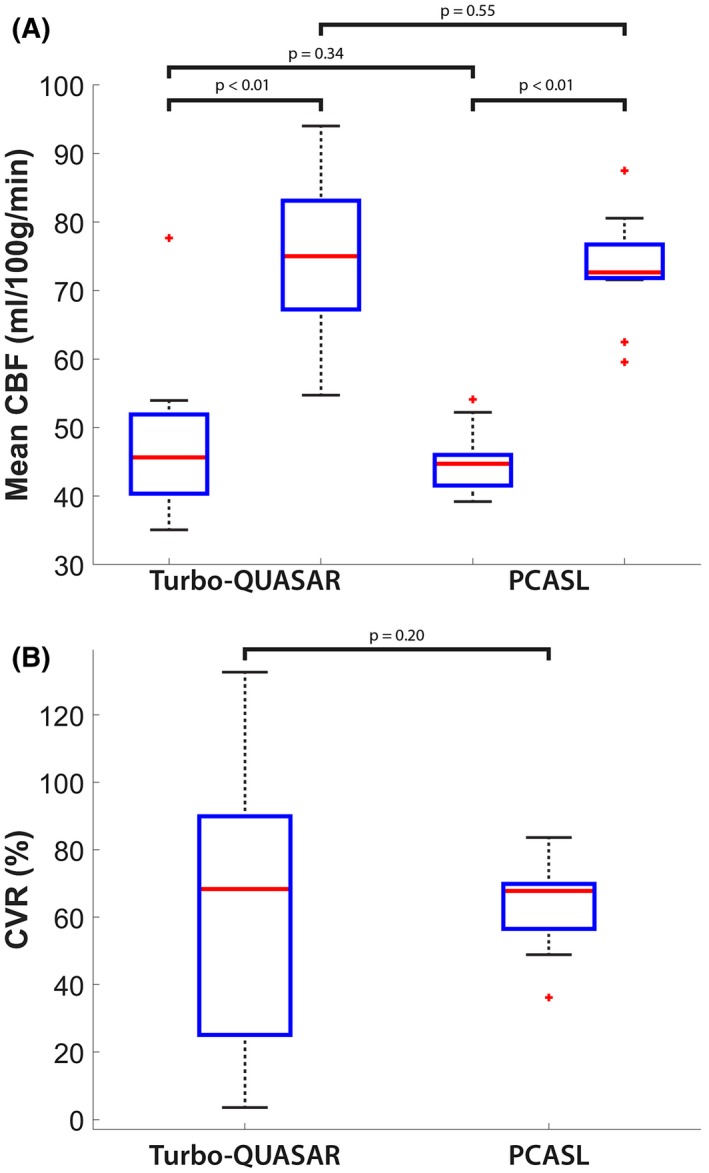
(A) Mean CBF of all subjects before and after acetazolamide administration. For all methods, the CBF increased significantly after acetazolamide administration. (B) Mean CVR of the group. For both ASL techniques, the mean CVR values of the group were between 63% and 64% but not significantly different from each other. Each box plot indicates the median, 25th, and 75th percentiles, maximum and minimum not considering outliers, and the outliers represented by the “+” symbol

Figure 3 shows the estimated CBF of an example subject using Turbo‐QUASAR and PCASL before and after acetazolamide administration. The slightly lower resolution of the Turbo‐QUASAR acquisition could be observed in the smoother appearance of the CBF maps in this case. Figure 4A shows the mean whole‐brain CBF of all subjects at baseline and after acetazolamide administration. Overall, both Turbo‐QUASAR and PCASL were sufficiently sensitive to detect significant CBF changes in this population after the administration of acetazolamide. At baseline, the mean CBF of the whole brain were between 45 and 50 mL/100 g/min for both methods. After the administration of acetazolamide, the mean CBF of the group increased significantly to between 75 mL/100 g/min and 77 mL/100 g/min for all methods. No significant CBF differences were observed between Turbo‐QUASAR and PCASL at both baseline and postacetazolamide conditions. The Turbo‐QUASAR difference data and the model‐fitting results of an example voxel in Turbo‐QUASAR data can be found in Supporting Information Figure S3.

**Figure 5:**
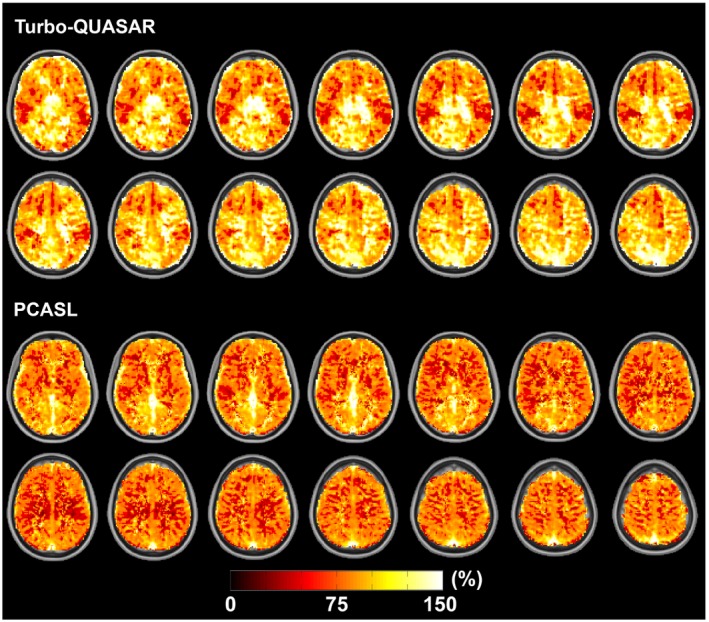
Estimated mean CVR map for each ASL method. In both Turbo‐QUASAR and PCASL results, the posterior region appeared to have the largest CVR. The views include transverse slices of z = 33, 35, …, 61

Figure 4B shows the mean whole‐brain CVR of the two ASL methods across the group, and Figure 5 shows the mean voxel‐wise CVR images. The mean CVR of the group was between 63% and 64% for the different ASL techniques, but they were not significantly different. For both ASL techniques, the CVR of the posterior regions of the brain appeared to be higher than the other regions.

**Figure 6:**
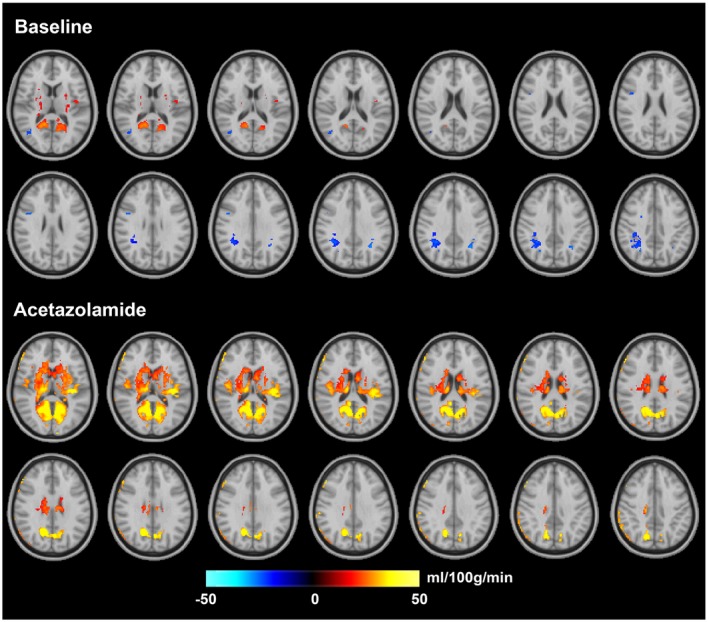
Regions of significant CBF differences between PCASL and Turbo‐QUASAR (*t* test; corrected *P* value <0.05) before and after acetazolamide administration. Hot color indicates that CBF of Turbo‐QUASAR is higher, and cold color indicates that CBF of PCASL is higher. The views include transverse slices of z = 33, 35, …, 61

Figure 6 shows the results of the paired *t* tests and the regional CBF differences (with corrected *P* value <0.05) between the two ASL methods before and after the administration of acetazolamide. Before the administration of acetazolamide, CBF of Turbo‐QUASAR in deep gray matter structures was significantly higher than the CBF of PCASL. The regional CBF differences increased after the administration of acetazolamide and were dominated by regions with higher CBF as estimated by Turbo‐QUASAR (hot colors).

**Figure 7:**
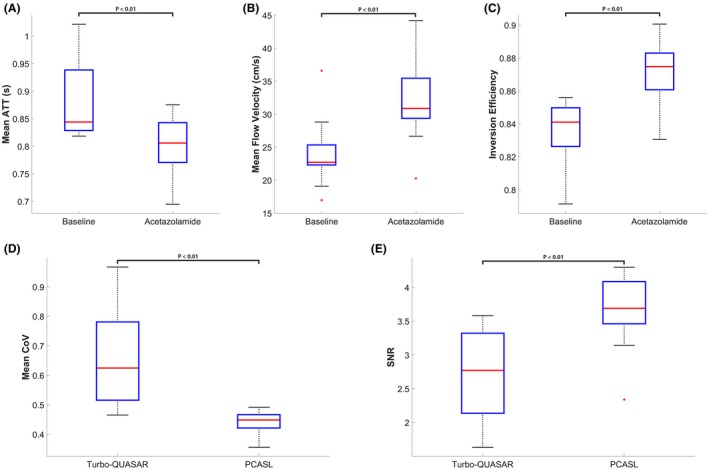
Estimated mean ATT from Turbo‐QUASAR, flow velocity from PC‐MRI, inversion efficiency of PCASL, and mean CoV of the two ASL techniques. (A) and (B) The administration of acetazolamide induced a significant decrease in mean ATT by 9.4% and a significant increase of mean flow velocity of the arterial blood in the internal carotid artery by 37%, and this led to (C) a significant increase in estimated inversion efficiency by 3.5%. (D) The mean CoV of the PCASL data was significantly lower than that of the Turbo‐QUASAR data by 34%. (E) Estimated SNR of Turbo‐QUASAR and PCASL. The SNR of PCASL was significantly higher than PCASL

Figure 7 shows the changes in the mean ATT estimated by Turbo‐QUASAR, changes in mean blood velocity in the internal carotid artery, resulting estimate of inversion efficiency for PCASL, mean CoV of the two ASL data sets, and estimated SNR of Turbo‐QUASAR and PCASL. Overall, the mean ATT decreased significantly by 10% postacetazolamide. In terms of the velocity in the internal carotid artery, the mean value for the baseline and acetazolamide conditions were 24.0 and 33.5 cm/s respectively, with a significant increase of 37%. From the Bloch equation simulations, this translated into a mean predicted increase in inversion efficiency from 0.84 to 0.88 for PCASL postacetazolamide. The increase in flow velocity also caused the mean SBD of Turbo‐QUASAR to decrease significantly from 0.58 to 0.46 seconds (21%) after the administration of acetazolamide. The estimated CoV of the Turbo‐QUASAR data was significantly higher (34%) than PCASL at baseline, implying that the estimated CBF of Turbo‐QUASAR had a higher variability and the CBF estimation from PCASL had a higher precision than the CBF estimated from Turbo‐QUASAR. The mean SNR of Turbo‐QUASAR was also significantly lower than that of PCASL, as shown in Figure 7E. The median across subjects of the SNR of PCASL (3.6) was significantly higher than Turbo‐QUASAR (3.1). Bland‐Altman plots showing the mean and difference between the CBF of Turbo‐QUASAR and PCASL can be found in Supporting Information Figure S4.

## 5 DISCUSSION

In this work, we have developed a model‐based method for estimating the hemodynamic parameters from Turbo‐QUASAR ASL and investigated its application for measuring CBF and CVR. The objective of the recently developed Turbo‐QUASAR technique was to include a frequent labeling scheme to achieve a higher SNR while retaining the measurement of ATT using a multi‐TI acquisition. In the simulations, the accuracy of the model‐based method for CBF estimation was evaluated to address the question of sensitivity to variation in bolus duration that might be brought about by changes in arterial blood velocity. We compared absolute CBF and CVR quantification from a healthy cohort data acquired using single‐PLD PCASL and Turbo‐QUASAR. The primary findings were: (1) The model‐based quantification method demonstrated good accuracy and consistency when the effective SBD is known, although fitting errors appeared if the true SBD was sufficiently shorter than expected; (2) both Turbo‐QUASAR and single‐PLD PCASL achieved similar whole‐brain CBF and CVR values; and (3) there were significant regional CBF differences in deep gray matter structures measured by the two ASL techniques.

### 5.1 Model‐based method and simulations for Turbo‐QUASAR

The model‐based method adopted here was accurate in estimating the hemodynamic parameters if the flow velocity of the arterial blood was normal (black curve of default SBD in Figure 2B), but it was limited by large deviations from the expected SBD value. If the SBD became shorter, such as might be expected under more rapid blood velocity in CVR studies, the estimation of all parameters (CBF, ATT, and SBD) became less accurate (default SBD in Figure 2B). The issue could be partially resolved by incorporating an estimated SBD in the model‐fitting procedure (corrected SBD in Figure 2B). This implies that where variation in arterial blood velocity is expected, it will be necessary to supply a separate estimate of the effective SBD for the accurate quantification of CBF. Further investigation indicated that the difficulty in simultaneously estimating CBF, ATT, and SBD with high accuracy is challenging because of the correlation between these three parameters in their effect on the resulting kinetic curve.

### 5.2 Comparison with PCASL in CBF quantification

In a healthy cohort, the full‐brain CBF estimated from the data collected by Turbo‐QUASAR was similar to that from single‐PLD PCASL (between 45 and 50 mL/100 g/min at baseline and 75 and 77 postacetazolamide). Although the results of the Turbo‐QUASAR method appeared to contain higher intersubject variations, the whole‐brain CBF was not significantly different from the results of PCASL in this population. The lower SNR (reflected by the higher CoV in Figure 7D) of Turbo‐QUASAR may be an important factor in explaining the higher intersubject variations observed in the Turbo‐QUASAR results. Despite the high variability in CBF quantification, Turbo‐QUASAR has the advantage over single‐PLD PCASL in that it can be used to measure ATT, as well as quantify the absolute CBF without acquiring a separate calibration data.^23^ The mean baseline ATT of the cohort was between 0.8 and 0.85 seconds (Figure 7A), in agreement with the literature.^4^ The CBF maps of Turbo‐QUASAR showed less contrast between gray and white matter than the results of PCASL, as seen in Figure 3. This may be a result of the lower spatial resolution of Turbo‐QUASAR (3.75 × 3.75 × 7 mm^3^) compared to PCASL (3 × 3 × 7 mm^3^), which would lead to more partial volume effects.

In the calibration of PCASL data, the partial volume effects in different brain tissues were not considered and a global $T_{1,tissue}$ of 1300 ms was assumed to correct the short TR of the M_0_ image following the work by Alsop et al.^3^ In reality, there will be partial voluming of white matter, gray matter, and cebrebrospinal fluid within the M_0_ image, which we have not attempted to correct for here. Correction is possible using partial volume estimates either derived from the T_1_‐weighted structural image, but reliant on a good registration, or from partial volume estimates made in the ASL space, for example derived from the saturation recovery of the Turbo‐QUASAR control images using the method in Zhao et al.^25^ This might alter some of the perfusion values noted in this study, but not the conclusions related to comparisons between PCASL and Turbo‐QUASAR.

Both before and after the administration of acetazolamide, a common feature of the regional CBF comparison was that there were no significant CBF differences for Turbo‐QUASAR and PCASL in cortical gray matter (Figure 6), where most ASL signal can be found. However, some regions of white matter and deep gray matter structures, which would have particularly prolonged ATT, showed significant differences, although the lower perfusion or SNR in the white matter makes this harder to interpret with confidence. It is possible therefore that the systematic differences observed between the two methods were associated with ATT. However, the size of the differences, which were of the order of 25 mL/100 g/min, would be larger than explained by ATT alone. Further investigation may be needed to resolve the CBF discrepancy in deep gray matter between Turbo‐QUASAR and PCASL.

ATT quantification has been investigated by several studies using different ASL techniques. In a study using multi‐PLD PCASL on healthy subjects, Qin et al reported a mean ATT of 1.4 ± 0.3 seconds for the whole brain,^30^ which overlapped with the range of ATT found in our study. A similar multi‐PLD PCASL technique has also been applied in a reproducibility study in which the researchers demonstrated a consistent measurement of ATT in the same session (interclass correlation = 0.908) using the same Variational Bayesian inference approach utilized in the present study.^31^ As for the ATT quantification after the administration of acetazolamide, although it is challenging to compare the present results with the literature because of the different vasoactive stimuli administered, a number of studies have been conducted to demonstrate the reduction in ATT after the administration of vessel dilation stimuli. For instance, Macintosh et al demonstrated a significant reduction in ATT by 12% after the administration of remifentanil using a multi‐TI PASL technique, reflecting the sensitivity of PASL to capture ATT variations attributed to vessel dilation.^32^ Donahue et al showed that regional ATT decreased between 4.6% and 7.7% measured by a multi‐PLD PCASL technique in hypercarbia.^7^

The comparison of the CBF maps of Turbo‐QUASAR and PCASL revealed that there was a significant difference in posterior brain regions (Figure 6), in the territory of the basilar artery. This may be a function of the effect of ATT, which is typically prolonged in this region compared to the rest of the brain and is prolonged further by the effects of dispersion.^33^ No attempt was made in the work to account for dispersion and the influence of cardiac pulsatility, which will potentially manifest in different ways in the quantification of CBF from both PCASL and Turbo‐QUASAR data. Although several bolus dispersion models have been developed for ASL techniques with a single labeling pulse,^18^, ^34^ a more comprehensive analysis is needed to investigate whether these models would be effective for the multiple sub‐boluses techniques used in Turbo‐QUASAR. Additionally, it remains possible that some differences between Turbo‐QUASAR and PCASL could arise from the macrovascular signal arising from labeled blood‐water in the larger arteries that has yet to be delivered to the tissue. This might be expected to be more prominent in PCASL at short PLDs and in more inferior slices where larger arteries are located. In theory, this is already directly corrected for as a source of contamination in the Turbo‐QUASAR analysis by using a combination of crushing gradients applied in different directions.^23^ Furthermore, the flow velocity of the vertebral arteries was different from carotid arteries, which may have implications for quantification for both PCASL and Turbo‐QUASAR, affecting both inversion efficiency and bolus duration.

In CBF quantification using Turbo‐QUASAR data, the SBD was estimated using the flow velocity measurement from PC‐MRI data. If the flow velocity is greater than the optimal value, a shorter SBD would be expected. If the velocity is less than the assumed value of the current implementation, a fraction of the sub‐bolus would still remain in the labeling region at the time of the next labeling pulse, leading to this part of the sub‐bolus being inverted twice and reducing the duration of the effective SBD. In the in vivo experiment, we have primarily focused our investigation on conditions in which a higher blood flow velocity was expected because the administration of acetazolamide increased the blood flow velocity. In the case of slower flow velocity, such as the blood near the vessel walls, the SBD would also be shorter than the optimal value. Although a potential extension to include the conditions of lower blood flow may be desired, it should be noted that the bolus dispersion issue would become more significant for the slow‐moving spins near the vessel walls and the inversion efficiency must be reconsidered because of the multiple inversion of the spins in the sub‐bolus.^18^ This is something that could be pursued using the same framework of this study in the future.

In the in vivo experiment using PCASL, a global ATT of 1300 ms was assumed for the CBF quantification. The PLD of the single‐PLD PCASL technique was specifically chosen to be insensitive to ATT variations within a reasonable range expected in the healthy cohort studied.^3^ Using the estimated ATT from the Turbo‐QUASAR data and accounting for differences between PCASL and PASL labeling by assuming a 500‐ms difference in ATT between the two techniques, we would estimate that a <1% difference in mean CBF might be observed attributable to not accounting for ATT in PCASL analysis. Such a discrepancy would reflect the previous findings by Alsop et al that CBF quantification should be fairly insensitive to ATT variations for PLDs longer than 1800 ms.^3^

### 5.3 Comparison with PCASL in CVR quantification

The CVR (Figures 4B and 5) and the statistical tests results (Figure 6) indicated that both ASL techniques were sufficiently sensitive to measure CVR. The measured mean CVR was between 63% and 64% for this population, which is within the range of the values measured by Positron Emission Tomography using ^15^O‐water as the tracer and acetazolamide as the stimulus.^35^, ^36^ Additionally, Turbo‐QUASAR offered the opportunity to measure changes in ATT with a mean reduction of 10% observed in this population (Figure 7A). Similar observations of a significant ATT decrease have been reported in CVR studies in which subjects experienced hypercarbia.^7^ Global CVR values measured by Turbo‐QUASAR demonstrated a higher intersubject variability than PCASL (Figure 4B), similar to the large intersubject variability noted in the CBF results (Figure 4A). Again, this might be explained by the lower SNR of Turbo‐QUASAR as indicated by the significantly higher CoV than PCASL (Figure 7D).

In quantifying the CVR values from PCASL data, the inversion efficiency was estimated using the PC‐MR data, and a significant increase (Figure 7C) was identified after the acetazolamide administration. Although such an observation may contradict with the belief that the inversion efficiency of PCASL should decrease for a higher flow velocity,^36^ the present study demonstrated that the velocity dependency of the inversion efficiency varied with the PCASL‐labeling parameters. The increase in flow velocity also caused the SBD of Turbo‐QUASAR to decrease significantly by 21%. Without accounting for such variations, the CBF and CVR of Turbo‐QUASAR would be 15% and 34% less (both statistically significant), respectively, than the current values after the administration of acetazolamide, leading to an underestimation of these parameters. Therefore, it is crucial to incorporate the flow velocity information for the accurate quantification of CBF and CVR.

### 5.4 Limitations

Throughout the study, we relied on the estimated flow velocity of the vessels that were segmented manually from the PC‐MRI data. The inversion efficiency of PCASL and the bolus duration of Turbo‐QUASAR were then estimated using the derived velocity values. Although the current results revealed that the inversion efficiency increased,^37^, ^38^ the sensitivity of the inversion efficiency to the estimated velocity remains to be more fully explored, in particular how variations in sequence parameters would affect the inversion efficiency. We have not sought to investigate all possible SBD variations induced by different flow velocities and have concentrated on the case in which the SBD was reduced because of a more rapid flow in the CVR experiment. A potential extension would be to consider the impact on Turbo‐QUASAR analysis attributable to slower blood flow. The range of velocity (from 0 to 100 cm/s) in computing the inversion efficiency of the PCASL analysis was chosen in order to ensure completeness and coverage of a wide range of velocity values. The narrower range of values used in the SBD of Turbo‐QUASAR reflected the more realistic values in this study. Although we primarily focused our investigation on these ranges of SBDs for the healthy cohort of this study, we believe that broadly the conclusion would hold based on the results of the simulation experiments.

## 6 CONCLUSIONS

In this work, we have developed a model‐based quantification technique for estimating hemodynamic parameters using Turbo‐QUASAR. The model‐based method demonstrated good accuracy and consistency in normal blood flow conditions in simulations, but it requires a separate estimate of the bolus duration when this deviates substantially from the normal value. For CVR quantification, both Turbo‐QUASAR and single‐PLD PCASL techniques achieved similar CVR measurements between 63% and 64% in a healthy population. Therefore, we can conclude that PCASL remains to be the favorable choice for CVR measurement because of its low CoV whereas Turbo‐QUASAR can serve as an alternative technique with the potential benefit of controlling for ATT variations.

## Supporting information


**FIGURE S1** Slice shifting strategy. In repeat 1, slices were acquired from bottom to top (slice A to D) in each TI, whereas in repeat 2, slices were acquired from middle to the top then from bottom to the middle (slice C to D then A to B). This effectively increases the number of slices acquired at each TI as well as the temporal resolution
**FIGURE S2** MT effects in Turbo‐QUASAR. Each curve shows the average signal of all the voxels in the slice of the corresponding color at different TI. From TI = 1 to TI = 6, the signal of the Turbo‐QUASAR control image is affected by both MT and Look‐Locker effects. After TI = 6, the signal is only affected by the Look‐Locker effect. The signal in the superior slice (blue) experiences less influence from the MT effects than the signal in the inferior slices (red) attributable to the different distance between the slice and the labeling location
**FIGURE S3** Turbo‐QUASAR difference data (middle slice) at each inversion time and model‐fitting results in an example voxel. Note that the data from the 2 TRs were combined using the slice‐shifting strategy to increase the effective temporal resolution from 11 to 22. The odd number slices were from the first TR, and the even number of slices were from the second TR. Overall, the ASL signal postacetazolamide was higher than the baseline signal, indicating an increase in CBF
**FIGURE S4** Bland‐Altman plots of the mean and differences of CBF before and after the administration of acetazolamide. In both plots, the solid line represents the mean difference between the CBF of Turbo‐QUASAR and PCASL. The dashed lines represent the 95% confidence interval of the mean difference
