# Supplementary material for: Quantification of cerebral perfusion and cerebrovascular reserve using Turbo‐QUASAR arterial spin labeling MRI
Source: Magn Reson Med. 2019 Sep 12;83(2):731–48. doi: 10.1002/mrm.27956 (PMC6899879; doi:10.1002/mrm.27956)
Supplement: Supplementary file 1 — FIGURE S1 Slice shifting strategy. In repeat 1, slices were acquired from bottom to top (slice A to D) in each TI, whereas in repeat 2, slices were acquired from middle to the top then from bottom to the middle (slice C to D then A to B). This effectively increases the number of slices acquired at each TI as well as the temporal resolution FIGURE S2 MT effects in Turbo‐QUASAR. Each curve shows the average signal of all the voxels in the slice of the corresponding color at different TI. From TI = 1 to TI = 6, the signal of the Turbo‐QUASAR control image is affected by both MT and Look‐Locker effects. After TI = 6, the signal is only affected by the Look‐Locker effect. The signal in the superior slice (blue) experiences less influence from the MT effects than the signal in the inferior slices (red) attributable to the different distance between the slice and the labeling location FIGURE S3 Turbo‐QUASAR difference data (middle slice) at each inversion time and model‐fitting results in an example voxel. Note that the data from the 2 TRs were combined using the slice‐shifting strategy to increase the effective temporal resolution from 11 to 22. The odd number slices were from the first TR, and the even number of slices were from the second TR. Overall, the ASL signal postacetazolamide was higher than the baseline signal, indicating an increase in CBF FIGURE S4 Bland‐Altman plots of the mean and differences of CBF before and after the administration of acetazolamide. In both plots, the solid line represents the mean difference between the CBF of Turbo‐QUASAR and PCASL. The dashed lines represent the 95% confidence interval of the mean difference [file MRM-83-731-s001.docx]

**Supporting Information**

In order to increase the effective temporal resolution, a slice shifting technique was adopted as illustrated in Supporting Information Figure S1. In the first repeat, the slices are acquired from most inferior to most superior, as in most 2D acquisitions in ASL. In the second repeat, the slices are acquired from the center to the most superior slice then from the most inferior slice to the center of the brain. Instead of taking the mean across all repeats as in conventional ASL, the signal from each repeat is rearranged such that each TI would contain a particular slice whose signal was acquired at a slightly different time as a result of the multi-slice acquisition. Thus, by interleaving the slices obtained from the two repeats, each TI would contain two acquisitions of the same slice and therefore double the effective temporal resolution.


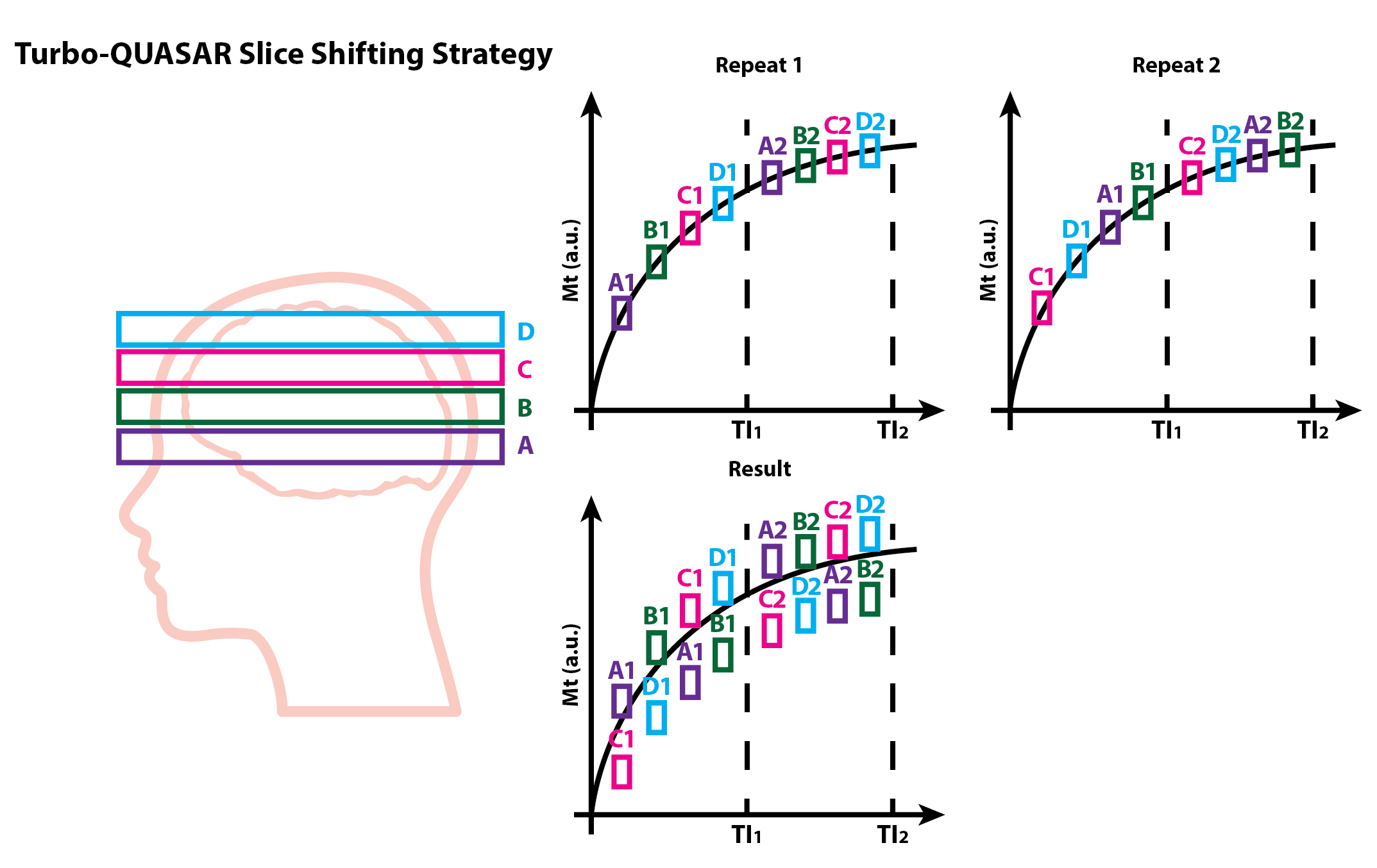


Supporting Information Figure S1: Slice shifting strategy. In repeat 1, slices were acquired from bottom to top (slice A to D) in each TI whereas in repeat 2 slices were acquired from middle to the top then from bottom to the middle (slice C to D then A to B). This effectively increases the number of slices acquired at each TI as well as the temporal resolution.


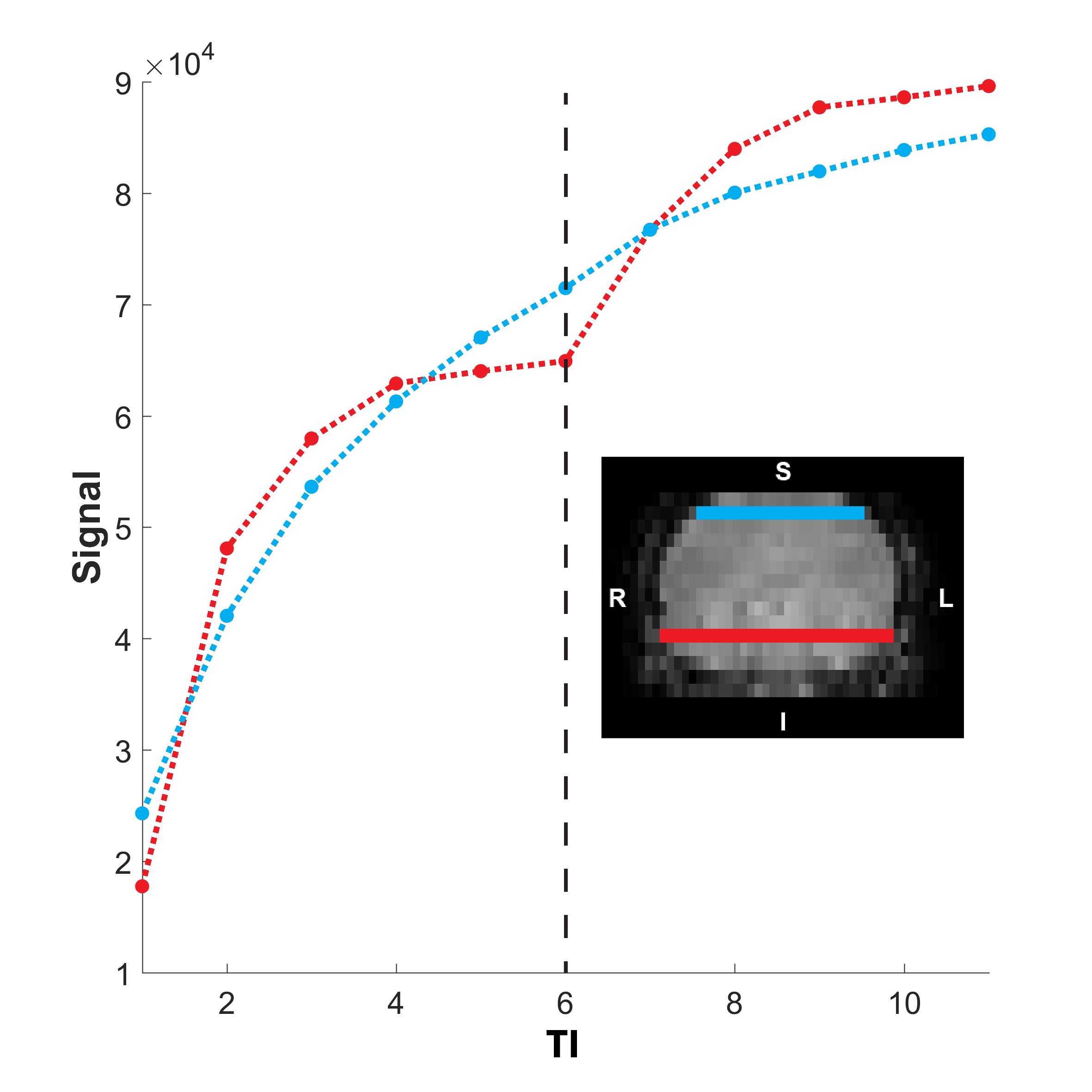


Supporting Information Figure S2: MT effects in Turbo-QUASAR. Each curve shows the average signal of all the voxels in the slice of the corresponding color at different inversion time (TI). From TI = 1 to TI = 6, the signal of the Turbo-QUASAR control image is affected by both MT and Look-Locker effects. After TI = 6, the signal is only affected by the Look-Locker effect. The signal in the superior slice (blue) experiences less influence from the MT effects than the signal in the inferior slices (red) due to the different distance between the slice and the labeling location.


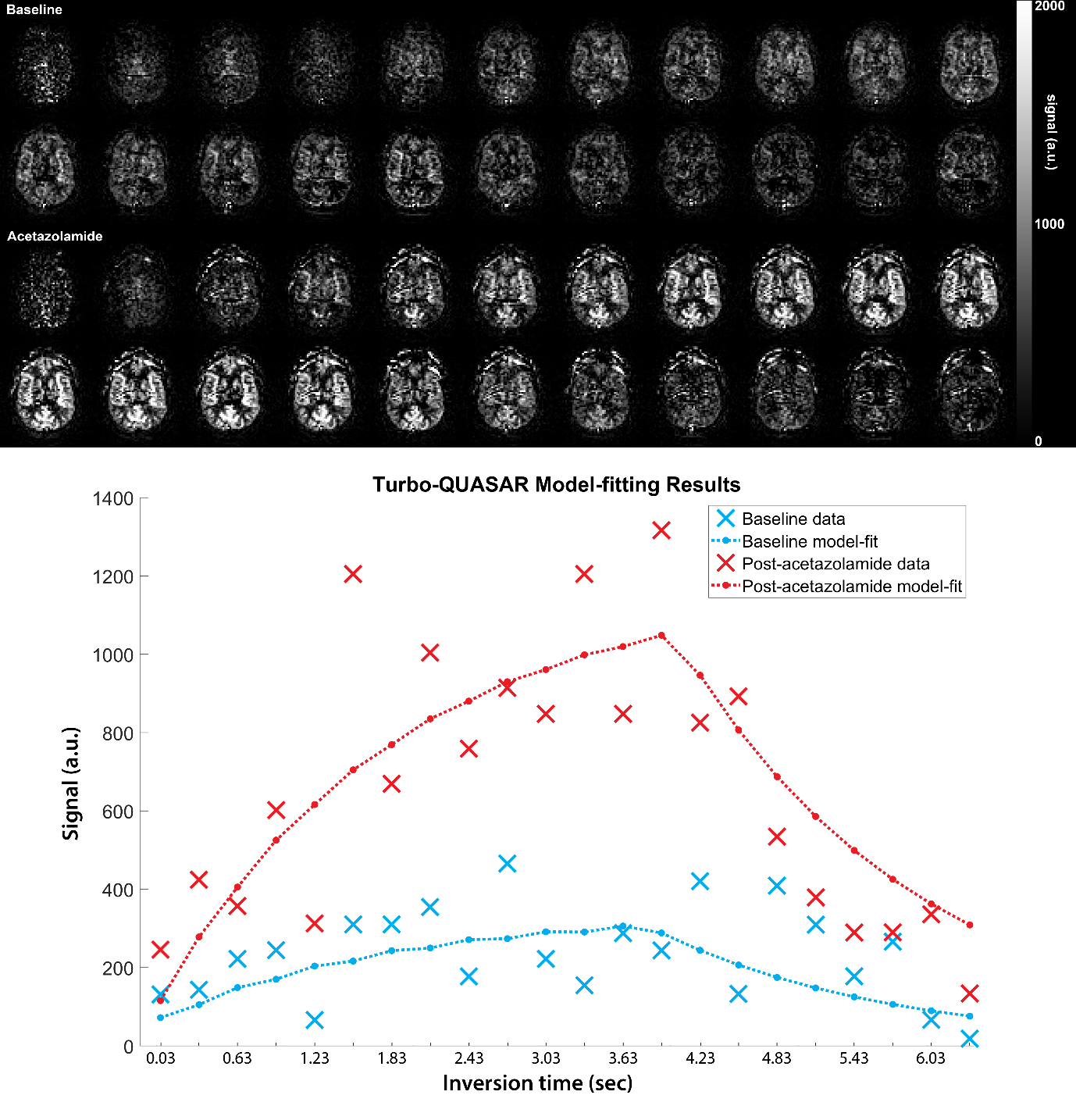


Supporting Information Figure S3: Turbo-QUASAR difference data (middle slice) at each inversion time and model-fitting results in an example voxel. Note that the data from the two TRs were combined using the slice shifting strategy to increase the effective temporal resolution from 11 to 22. The odd number slices were from the first TR and the even number of slices were from the second TR. Overall, the ASL signal post-acetazolamide was higher than the baseline signal, indicating an increase in CBF.


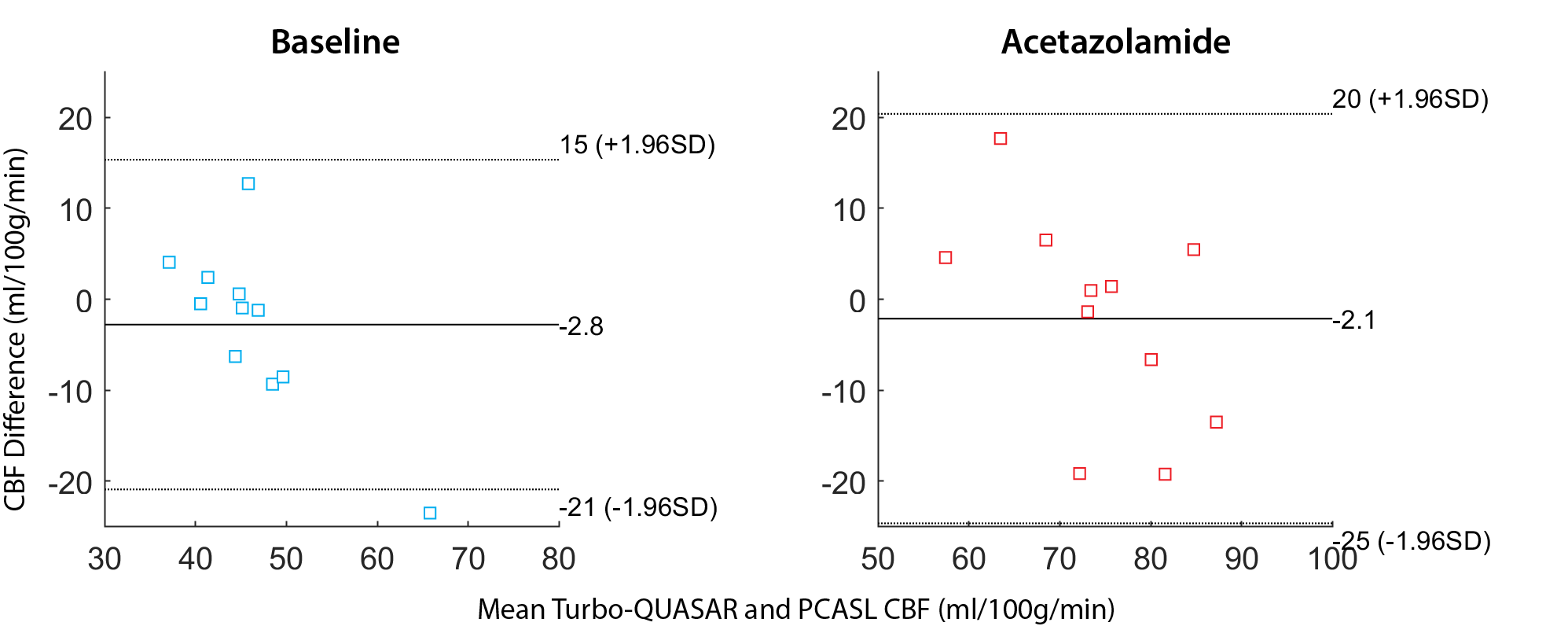


Supporting Information Figure S4: Bland-Altman plots of the mean and differences of CBF before and after the administration of acetazolamide. In both plots, the solid line represents the mean difference between the CBF of Turbo-QUASAR and PCASL. The dashed lines represent the 95% confidence interval of the mean difference.
